# Pyomyositis is not only a tropical pathology: a case series

**DOI:** 10.1186/s13256-016-1158-2

**Published:** 2016-12-21

**Authors:** Laura Comegna, Paola Irma Guidone, Giovanni Prezioso, Simone Franchini, Marianna Immacolata Petrosino, Paola Di Filippo, Francesco Chiarelli, Angelika Mohn, Nadia Rossi

**Affiliations:** 1 Department of Paediatrics, University of Chieti, Chieti, Italy; 2 University “G. D’Annunzio”, Chieti, Italy

**Keywords:** Pyomyositis, Muscle infection, Childhood, Case report, Tropical pathology

## Abstract

**Background:**

Pyomyositis is an acute bacterial infection of skeletal muscle that results in localized abscess formation. This infection was thought to be endemic to tropical countries, and is also known as “tropical pyomyositis”. However, pyomyositis is increasingly recognized in temperate climates and is frequently associated with an immunosuppressive condition, such as human immunodeficiency virus, malignancy, and diabetes mellitus. It is also found in healthy and athletic people after strenuous or vigorous exercise or following localized and possibly unnoticed trauma. It can be primary or secondary to neighboring or remote infection. Primary pyomyositis is a rare condition that can affect children and adolescents. Diagnosis can be delayed because the affected muscle is deeply situated and local signs are not apparent. This delay in diagnosis can result in increased morbidity and a significant mortality rate. The pediatric population, which comprises 35% of the reported pyomyositis cases, is an especially difficult subset of patients to diagnose.

**Case presentation:**

In our series, we describe the cases of four previously healthy Caucasian children who were admitted to our Pediatric Department with different clinical presentations. Pyomyositis in our patients was related to factors affecting the muscle itself, including strenuous exercise and direct muscle trauma. Therapy was started with a cephalosporin antibiotic and teicoplanin was subsequently added. The minimum length of therapy was 3 weeks.

**Conclusions:**

The diagnosis of pyomyositis in our patients, none of whom were immune-compromised, is confirmation that this disease is not an exclusive pathology of tropical countries and demonstrates that there is an increasing prevalence of pyomyositis in temperate climates.

## Background

Pyomyositis is a bacterial infection that results in an intramuscular abscess of the large skeletal muscle groups. The first case was documented by Scriba in Japan in 1885 [1] but the first report in North America did not appear until 1971 [2]. This infection is endemic to tropical countries, accounting for 2.2–4% of surgical admissions [1]. It is much less common in temperate climates, such as North America, where only 98 cases were reported between 1972 and 1992 [3]. In tropical countries, peak incidence is at 2–5 years of age; in North America, the mean age is 8.4 years with a range of 1–16 years [1].

Primary pyomyositis is common in many parts of Africa and in the South Pacific, and has earned the name “tropical pyomyositis.” It occurs more frequently in immune-compromised patients, though it can be related to factors affecting the muscle itself (strenuous exercise, direct muscle trauma, viral or parasitic myositis) [4]. Secondary pyomyositis is usually a consequence of direct extension from an infectious process, most notably Crohn’s disease, infectious colitis, appendicitis, and neoplasia. The pathogenesis of the disease is not clear, but trauma, malnutrition, viral and parasitic infections, bacteremia, immunodeficiency or chronic illness, and other factors may have predisposing roles [1]. Intensive exercise and local trauma have been suggested as risk factors, but only a third of patients had evidence of these risk factors [5]. Because of its rarity and often vague clinical presentation, it is unlikely to be considered during an initial differential diagnosis. Moreover, the diagnosis can be delayed because the affected muscle is deeply situated and local signs are not apparent. This delay in diagnosis can result in increased morbidity and a significant mortality rate [6]. The pediatric population, which comprises approximately 35% of reported cases, is an especially difficult subset of patients to diagnose [7].

**Fig. 1:**
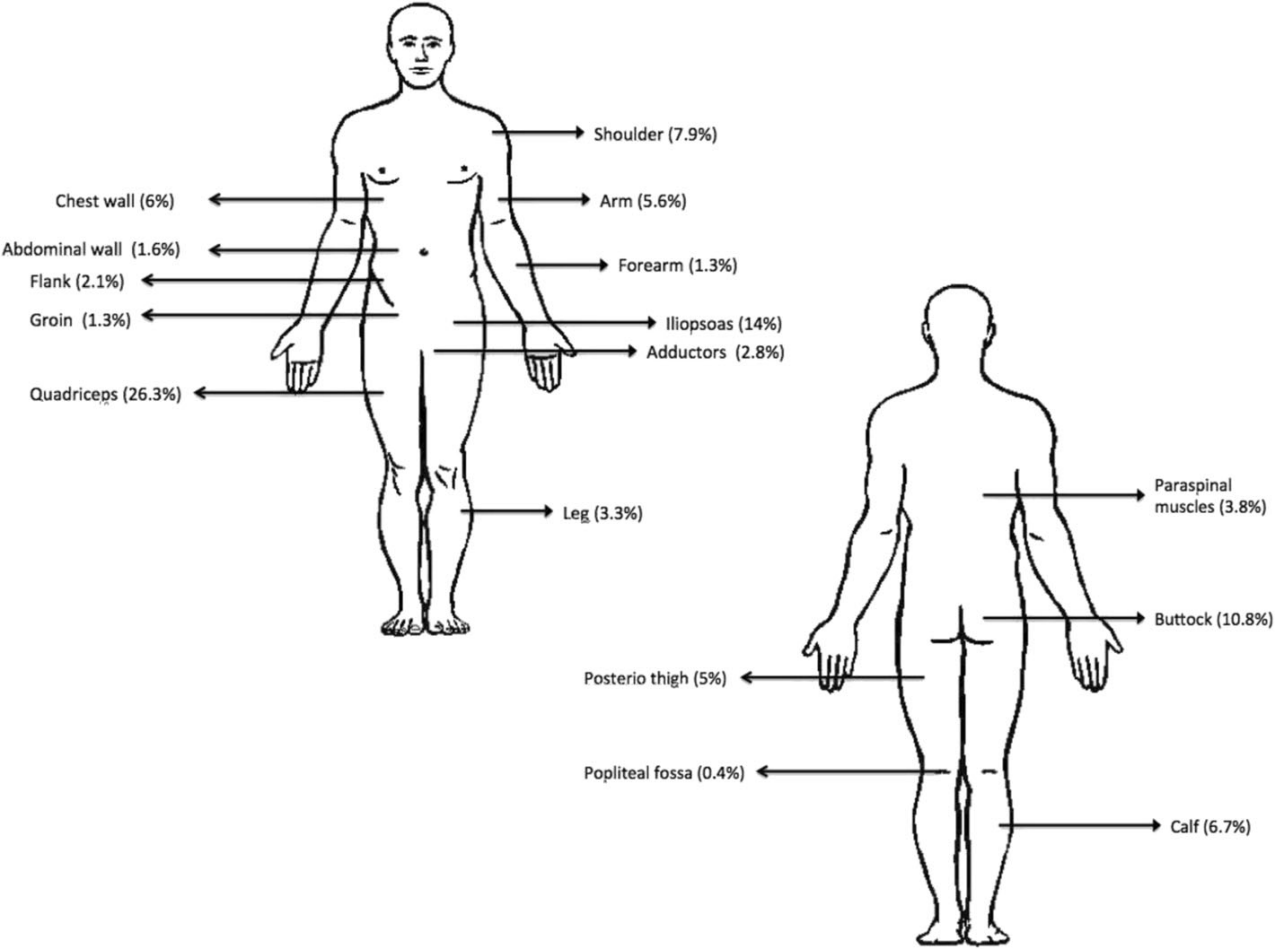
Anatomic distribution of pyomyositis modified from Bickels *et al.* [8]

Pyomyositis predominantly affects the muscles of the lower limbs, although it can also involve muscles of the upper limb, trunk, and spine (Fig. 1). In the lower limb, the most commonly affected muscles are the quadriceps and iliopsoas followed by the gluteal muscles. Pyomyositis originating in the obturator internus or/and external muscles is exceptionally rare; it occurs most commonly in children and young adults and exhibits a 2:1 prevalence in male patients [6].

If the disease is recognized early, antibiotic therapy alone is usually sufficient. Initial therapy should include a broad-spectrum agent with adequate *Staphylococcus aureus* coverage, and therapy should subsequently be modified depending on results of blood culture and sensitivity tests. Coverage of Gram-negative organisms should be empirically included in the treatment of immunocompromised patients. The therapy is initially administered intravenously and generally lasts 3–4 weeks but the duration can vary considerably [5].

## Case presentation

### Case 1

A 6–5/12-year-old Caucasian boy was admitted to our Pediatric Department with fever, pharyngitis, lateral cervical and inguinal lymphadenomegaly, limp, and pain in his proximal right thigh (medial side) during walking and on palpation. Prior to admission, he had a 12-day history of intermittent-remittent fever up to 39.3 °C, treated with paracetamol (acetaminophen). At admission, his C-reactive protein (CRP) concentration was 7.17 mg/dl (normal value <0.5 mg/dl) and he had leukocytosis (white blood cells (WBC) 19.36 × 10^3^/μl) and neutrophilia (15.77 × 10^3^/μl; 81.4%). For this reason, intravenous antibiotic treatment with ceftriaxone was started. Right lower limb venous ultrasonography excluded phlebitis or phlebothrombosis. Nevertheless, his CRP concentration increased to 15.90 mg/dl, his erythrocyte sedimentation rate (ESR) was 51 mm/h (normal value <20 mm/h), and there was a further increase in his leukocytosis (WBC 22.30 × 10^3^/μl) and neutrophilia (17.94 × 10^3^/μl; 80.5%); his antistreptolysin O (ASO) titer was 451 UI/ml (normal value <200 UI). Ultrasonography of his thigh muscles and hip was performed, showing a minimal fluid intrafascial thickening of his proximal adductor muscle. Double antibiotic therapy with ceftriaxone and teicoplanin was therefore started. Magnetic resonance imaging (MRI) with contrast confirmed the proximal thickening of his right proximal longus and magnus adductor muscles and ipsilateral proximal gracilis muscle, attributable to an inflammatory process, without any involvement of his joints. Results from subsequent laboratory tests showed a reduction in inflammatory markers (CRP 0.77 mg/dl, ESR 14 mm/h), an improvement in leukocyte (13.99 × 10^3^/μl) and neutrophil (9.07 × 10^3^/μl; 64.8%) concentrations, and normalization of his ASO titer. On the eighth day of treatment, the ceftriaxone therapy was interrupted and teicoplanin was administered for a further 7 days. At discharge, after normalization of his laboratory test results, oral therapy with amoxicillin plus clavulanic acid was started for an additional 7 days. A control MRI scan highlighted a nearly complete resolution of the pyomyositis in both muscles with a regression of fever, limp, and pain. The length of therapy was 3 weeks.

### Case 2

A 15-year-old Caucasian boy presented to our Pediatric Department with pain in his right buttock that began a week after a 3-km run and a bowling match, with radiation of the pain in his lumbar region that was aggravated by movement and an inability to walk unaided. There were no associated abdominal or urinary symptoms. Before admission, he had fever up to 39 °C. A physical examination showed fever (38.2 °C), right low back pain, a positive result on a leg raise test, and functional limitation in his right lower limb. He had some discomfort in his right groin on deep palpation to his right buttock lateral to the ischial tuberosity. Our patient was unable to ambulate independently but his muscle tone, strength, and reflexes were preserved. No sensory deficit of his right lower limb was noted. There ere no palable inguinal lymph nodes.

Initial laboratory studies revealed leukocytosis and neutrophilia (15 × 10^3^/μl; 90%), a CRP concentration of 15.80 mg/dl (normal value <0.5 mg/dl), and an ESR of 54 mm/h (normal value <20 mm/h). Involvement of the lower limb nerve was suspected. Routine radiography of both his hips and pelvis demonstrated no abnormalities. He was started on diclofenac for the pain and oral antibiotic treatment with amoxicillin plus clavulanic acid. An MRI of his lumbosacral spine and pelvis was performed; the examination showed an increased silhouette on his right side associated with areas of altered signal in the iliopsoas muscle, obturator, and gluteus medius and maximus. Some fluid collection (the largest of which measured up to 6 cm on the craniocaudal diameter), edema between the paraspinal muscles at L4–L5, and bilateral edema of the plan of dermal-subcutaneous thigh root were also reported in the posterolateral view. The antibiotic therapy was therefore replaced with ceftazidime and teicoplanin. A blood sample, taken on the 14th day of antibiotic therapy, showed a decrease in inflammatory markers (CRP 2.41 mg/dl, ESR 14 mm/h) and in neutrophilic leukocytosis (WBC 12.6 × 10^3^/μl, neutrophils 8.5 × 10^3^/μl). A control MRI scan highlighted a slight reduction in the fluid collections in his iliopsoas and obturator muscles, with the fluid collection in his gluteal muscles converted into an abscess, characterized by a mildly hyperintense labrum on T1-weighted MRI. At discharge, after normalization of his laboratory test results, oral therapy with amoxicillin plus clavulanic acid was started for an additional 4 weeks. The total length of therapy was 6 weeks.

### Case 3

An 8-year-old Caucasian boy presented to our Pediatric Department with a 1-week history of fever, pain, and impairment in walking owing to left leg pain. Before admission, he was treated with cephalosporin (ceftriaxone), acetaminophen, and ketoprofen without benefit. No association with trauma or previous infection was reported, but a knee injury was observed. A physical examination showed antalgic positioning of his left hip, warmth and pain on palpation of the proximal posterior region of his left leg, and stiffness and pain during leg movements. Neurological, respiratory, and cardiovascular examinations were normal. Laboratory tests documented an increased CRP concentration (6.25 mg/dl, normal value <0.5 mg/dl), ESR (72 mm/h, normal value <20 mm/h), leukocyte count (WBC 15.85 × 10^3^/μl), and creatine phosphokinase (CPK) (237 U/L). He had positive titer tests for ASO (648 UI/ml) and anti-staphylococcal antibodies. Owing to the suspicion of a *S. aureus* infection, a double therapy with cephalosporin and glycopeptide antibiotics (ceftriaxone plus teicoplanin) was undertaken for 8 days. A hip X-ray documented reactive changes in the lesser trochanter of his femur and an ultrasound scan showed a hematoma at the proximal third of the posterior side of his thigh. MRI showed inflammation of his obturator and adductor magnus muscles. Our patient was discharged on oral antibiotic therapy for 10 days. The follow-up visits documented a gradual resolution of symptoms, normal laboratory test results, and reduced muscle inflammation in an MRI scan. No drainage was necessary. The length of therapy was 3 weeks. A few months later our patient was symptom free.

### Case 4

A 10–6/12-year Caucasian girl presented to our Pediatric Department with pain localized to her right thigh for 8 days’ duration, with significant movement restriction, an inability to bear weight, and fever (up to 39.7 °C). Our patient had been treated at home with anti-inflammatory therapy (ibuprofen). Normal results were seen on ultrasonography of her right hip and on right pelvic and hip radiography. On examination, our patient had an attitude of flexion and adduction in her right hip, evident restriction of her right lower limb movements, and painful passive mobilization. The pain worsened when the proximal adductor region was palpitated. There was also swelling over the medial side of the root of her thigh. Laboratory studies showed elevated CRP levels (14.4 mg/dl, normal value <0.5 mg/dl), a slight increase in lactic dehydrogenase values (712 U/l), and neutrophilia (10.7 × 10^3^/μL; N: 7:57 × 10^3^/μL). Her ESR was not measured in the emergency room. Septic arthritis was suspected and intravenous antibiotic therapy with ceftazidime and anti-inflammatory therapy with ibuprofen were started. MRI of her pelvis and lumbosacral spine demonstrated intra-articular effusion of her right hip joint with short TI inversion recovery (STIR) hyperintensity in her ipsilateral adductor muscles. Follow up blood tests documented a progressive reduction of CRP (1.29mg/dL); ESR: 47 mm/h) and normalization of her WBC count (7.12 × 10^3^/μL). Results from a Widal-Wright test and blood culture were negative. Results from a urine analysis were normal. Owing to a positive anti-staphylococcal antibody titer, teicoplanin was added to her ceftazidime therapy. During her hospitalization, our patient’s clinical condition progressively improved: her fever disappeared in 10 days with remission of the painful limitation to active and passive motions of her right hip after 12 days, and subsequent recovery of ambulation. Our patient was discharged with an oral course of amoxicillin-clavulanate treatment, thus completing a 3-week cycle of antibiotic therapy. A second MRI was performed after therapy completion, which showed nearly complete resolution of the inflammatory changes. At a 1-month follow-up, our patient was symptom free without sequelae.

## Discussion

The clinical presentation of pyomyositis in our series was varied. Results from a physical examination are usually not clear and the diagnosis is often delayed. A mean delay of 10 days from the onset of the symptoms to correct diagnosis has been reported in the literature [8]. Some authors have outlined three stages of pyomyositis as a gradual progression from diffuse inflammation to focal abscess formation in a septic state (Table 1) [1–3, 5, 6, 9–12]. In our series, all patients were at stage 2.

**Table 1:** Clinical stages of pyomyositis [1–3, 5, 6, 8–11]

Stage 1	Invasive	About 2% of patients present in this stage. This stage lasts from 10 to 21 days. Insidious onset of dull, cramping pain, a low-grade fever, muscle ache, general malaise, and anorexia. There is localized edema, sometimes described as indurated or woody, with little or no tenderness. There is no abscess, and examination of aspiration specimens is unrevealing.
Stage 2	Purulent or suppurative	About 90% of patients present in this stage. In one study, the mean duration of symptoms before hospitalization was 24 days, whereas in other studies it was respectively 12 days and 6.2 days. Fever and chills. This phase is characterized by abscess formation. Pathologic findings on biopsy show edematous muscle fibers, lymphocytic infiltration, and suppuration as the muscle belly is replaced by pus. The involved muscle is usually tender and the overlying skin may be normal or mild eruptive.
Stage 3	Late stage	High fever, severe pain, local signs of infection and systemic manifestations of sepsis may be present. The final stage is characterized by septicemia, metastatic abscesses, and multi-organ dysfunction, and is associated with high mortality. There is exquisite tenderness of the involved area.

Laboratory investigations are not specific and are rarely helpful in the diagnosis of pyomyositis [8]. In our series, we reported the same laboratory findings as found in previous studies, such as moderate leukocytosis with a left shift, elevation of ESR and CRP, and normal muscle enzyme levels [1], although an increase in CPK was also documented in case 3. Three cases involved an elevated ASO titer and two had positive staphylococcal antibody titers. Imaging is the best modality to define the features and the extent of muscle infection.

Various noninvasive diagnostic modalities such as ultrasound, computed tomography (CT), and MRI have been used to evaluate patients with suspected musculoskeletal infection [13]. Plain radiography is appropriate for the initial screening [8]. Only a minority of patients have findings suggestive of muscle inflammation. X-rays was performed only in case number 3 and it failed to document the specific reactive change in the adjacent bone. A CT scan provides better delineation of muscle than X-rays and can identify a muscle abscess, but it may fail to demonstrate inflammatory changes in earlier stages [8]. In our series, no CT scans were performed when ultrasonography was used. The typical findings on radiography are a bulky muscle with abnormal echotexture and a hypoechoic focal lesion, occasionally with internal debris and air bubbles [8, 14]. In our cases, ultrasonography allowed visualization of significant muscle inflammation. MRI is the gold standard, showing the extent of involvement and the site of fluid collection. It offers the additional advantage of being able to study the adjacent structures, such as joints, bone, and soft tissue [15], and is therefore helpful to differentiate other pathological processes from pyomyositis [12], such as infectious arthritis of the hip, osteomyelitis, hematoma, or soft-tissue tumor [8]. All four of our patients underwent MRI, which was very helpful in defining the appropriate therapy. In three cases, MRI was also repeated to evaluate the resolution of inflammation. Technetium or gallium scintigraphy is rarely used in the diagnosis of pyomyositis, but it may assist the diagnostic process when the clinical presentation is equivocal [8].

Clinical manifestations of pyomyositis are varied so it is very important to make an early and correct diagnosis and to exclude other pathologies (Table 2).

**Table 2:** Symptoms and differential diagnosis [1, 10, 15, 18–22]

Specific site of tenderness and swelling	Osteomyelitis, septic arthritis [1]
Fever and abdominal pain	Acute abdomen or basal lobe pneumonia [15, 18]
Muscular hematoma, rupture, or strain	Trauma [1]
Localized muscular mass, painless slow-growing masses, painful mass increasing in size	Sarcoma [19, 20]
Pain in right lower quadrant, back, flank, or hip; fever	Acute appendicitis or acute disease of hip or femoral nerve [21]
Myalgia, weakness, tea-colored urine	Rhabdomyolysis [14, 22, 23]

The choice of treatment is usually related to the stage of disease at presentation. Prompt, accurate diagnosis followed by early initiation of intravenous antibiotics can prevent abscess formation and avoid surgical drainage [10]. In the early invasive stage, in the absence of a drainable collection of purulent fluid on MRI, diffuse inflammation can be treated empirically with antibiotics alone [16]. On the basis of the most likely culpable bacteria, cloxacillin is a common choice. A combination of cloxacillin and aminoglycoside achieves a synergic effect for septic or immunocompromised patients [8]. Most patients with primary pyomyositis can be treated successfully with intravenous administration of a single antibiotic. In our series, the therapy was started empirically with a double antibiotic therapy with cephalosporin and glycopeptide antibiotics (ceftriaxone, cefixime, or ceftazidime plus teicoplanin) for at least 2 weeks to cover *S. aureus* and Gram-negative infection. Antibiotic therapy was discontinued when physical signs and symptoms disappeared and blood test results and CRP concentration returned to normal values. The duration of therapy has not been established and can vary from 2 to 6 weeks, depending on clinical severity and the patient’s response. Patients who have an underlying disease that compromises their immunity may require a longer period of intravenous and oral antibiotic treatment [8].

Abscess formation, however, requires appropriate drainage before starting antibiotics. Because most patients are not seen until an abscess has already formed (stages 2 and 3), drainage followed by intravenous administration of at least two broad-spectrum antibiotics remains the treatment of choice [8]. When the pathogenic agent is isolated, antibiotic therapy should be adjusted according to the result of culture and sensitivity tests [10]. In many series, despite abscess formation in some patients, neither surgical nor percutaneous drainage are needed. Traditionally, abscesses were drained by operative means, which entailed a large skin incision and a wide exposure of the affected muscle. Similarly, iliopsoas pyomyositis necessitated laparotomy and extensive retroperitoneal exposure. Following operative drainage, packing and daily dressing changes were necessary to prevent the abscess cavity from closing prematurely [17]. However, now drainage can be accomplished percutaneously under ultrasound or CT guidance, and operative intervention is used only when complete drainage cannot be achieved percutaneously. Streptococcal pyomyositis is associated with extensive muscle necrosis that requires not only abscess drainage, but also radical excision of all necrotic tissues. When there has been a delay in diagnosis, a more extensive debridement and, rarely, amputation may be needed [8]. A diagnostic delay could lead to sepsis and death [15], so it is very important to choose the correct diagnostic approach.

## Conclusions

Pyomyositis in our series was related to factors affecting the muscle itself, including strenuous exercise and direct muscle trauma. The gold standard for diagnosing pyomyositis is MRI. Ultrasonography is useful for monitoring muscle inflammation. In our series, therapy was started with a cephalosporin antibiotic and teicoplanin was subsequently added to cover *S. aureus* infection. The minimum length of treatment was 3 weeks. Our patients, none of whom were immune-compromised, lived in a temperate climate, confirming that pyomyositis is not an exclusive pathology of tropical countries.
